# A feasibility pilot trial of a peer-support educational behavioral intervention to improve diabetes medication adherence in African Americans

**DOI:** 10.1186/s40814-022-01198-7

**Published:** 2022-11-14

**Authors:** Olayinka O. Shiyanbola, Martha Maurer, Mattigan Mott, Luke Schwerer, Nassim Sarkarati, Lisa K. Sharp, Earlise Ward

**Affiliations:** 1 Division of Social and Administrative Sciences, University of Wisconsin-Madison School of Pharmacy, Madison, WI USA; 2 Sonderegger Research Center, School of Pharmacy, University of Wisconsin-Madison School of Pharmacy, Madison, WI USA; 3 School of Nursing, University of Wisconsin-Madison, Madison, WI USA; 4 School of Pharmacy, University of Wisconsin-Madison, Madison, WI 53705 USA; 5 Department of Pharmacy Systems, Outcomes and Policy, University of Illinois, Chicago, IL USA

**Keywords:** Diabetes, African Americans, Peer support, Medication adherence, Health beliefs/misinformation, Self-efficacy

## Abstract

**Background:**

African Americans are twice as likely to die from diabetes, compared to other racial and ethnic groups in the USA. Poor adherence to diabetes medications is common among African Americans and contributes to these disproportionally worse outcomes. A pilot study was conducted to determine the feasibility and acceptability of a peer-supported intervention targeting diabetes and medication beliefs, communication, and self-efficacy skills to enhance medication adherence among African Americans with type 2 diabetes.

**Methods:**

Based on the extended self-regulatory model and information-motivation-behavioral skills model, this intervention was piloted using a single group pre/post-intervention study design at two sites. Seventeen African Americans who self-reported as adherent to diabetes medicines (ambassadors) were paired with 22 African Americans with self-reported poor medication adherence (buddies). Feasibility outcomes evaluated recruitment, retention, and intervention adherence. Measures assessed at baseline and 1-month post-intervention included glycemic control (hemoglobin A1c), self-reported medication adherence, diabetes beliefs, concerns about diabetes medicines, and diabetes self-efficacy. Wilcoxon signed-rank tests assessed for differences in mean scores of outcome variables at baseline compared with a 3-month follow-up. Semi-structured 60-min interviews were conducted with each buddy to explore their acceptability of the intervention. To ensure the rigor of the qualitative data, we focused on analytic criteria such as credibility, confirmability, and transferability.

**Results:**

Most buddies and ambassadors were female and about 56 years old. Feasibility outcomes included recruitment success rates of 73% for buddies and 85% for ambassadors relative to our goals. Retention rate for hemoglobin A1c and medication adherence outcome assessment was 95% for buddies. Both buddies and ambassadors had excellent intervention adherence, with buddies having a mean attendance of 7.76 out of 8 sessions/phone calls and ambassadors completing > 99% of the 105 intervention calls with Buddies. Results showed a signal of change in hemoglobin A1c (effect size = 0.14) and medication adherence (effect size = 0.35) among buddies, reduction in buddies’ negative beliefs about diabetes and an increase in necessity beliefs of diabetes medicines. Summative interviews with buddies showed they valued ambassador’s encouragement of self-management behaviors.

**Conclusions:**

Results support conduct of an efficacy trial to address medication adherence for African Americans with type 2 diabetes using a peer-supported tailored intervention.

**Trial registration:**

https://clinicaltrials.gov/ct2/show/NCT04028076.

## Key messages regarding feasibility

- It is not known if a peer-supported intervention to address diabetes medication adherence among African Americans is feasible.
- Findings show good feasibility and acceptability of the intervention.
- Results support conduct of an efficacy trial using the intervention.

## Background

The prevalence of type 2 diabetes is twice higher in African Americans compared to non-Hispanic Whites [1]. As well, the rates of complications due to diabetes including deaths are higher in African Americans compared to other racial and ethnic groups. For example, death rates due to diabetes is much higher among African Americans compared to non-Hispanic Whites, and kidney disease, blindness, and amputations occur four times more among African Americans compared to other racial and ethnic minority groups [2]. Diabetes self-management behaviors including medication adherence remain a challenge for African Americans possibly due to culturally influenced health beliefs that do not align with care. Therefore, culturally tailored care that enhances patients’ adherence to treatment recommendations is important in reducing diabetes complications [3–5].

African Americans and other racial and ethnic minoritized groups are known to have poorer treatment adherence compared to non-Hispanic Whites [6]. Shenolikar et al., 2006, reported that African Americans with type 2 diabetes had 12% lower medication adherence compared to whites, which may have led to more diabetes-related morbidity [4]. A possible reason for the disparities in medication adherence and poorer outcomes compared to Whites is the differences between patients and provider perceptions of illness and medicines [7]. As well, there may be underlying factors such as patient beliefs and practices or low provider engagement which impact patient-provider communication and low patient medication self-efficacy [4, 8]. These psychosocial factors, defined as “characteristics that influence an individual psychologically and/or socially,” describe individuals in relation to their social environment and how such characteristics may affect their physical and mental health [9]. Because of this, it is important for medication adherence interventions to address pertinent patient psychosocial factors such as beliefs in illness and medicines, self-efficacy, and provider communication which are critical in reducing health disparities [10–12].

Medication adherence can be improved by intervening to address illness and medication beliefs [13–16]. Studies show that beliefs, social support, and self-efficacy influence treatment adherence for African Americans and may be targeted in tailored interventions [17, 18]. More so, prior interventions focused on reframing misbeliefs about disease and health behaviors and increasing self-efficacy have been effective among African Americans [19, 20]. Hence, we developed and implemented an intervention that targeted these factors to improve diabetes medication adherence for African Americans.

Diabetes self-management is a physical and emotional task for patient [21–23]. While providing social support to patients can be beneficial, previously used approaches such as nurse and health professional staff making phone calls to patients are time-and resource intensive [24, 25]. Peer supporters are an informal, flexible, and low-cost means of providing peer support and have provided great benefits to patients with diabetes [26–29]. One-on-one peer interactions between individuals with diabetes provide emotional and informational support, allowing for mutual reciprocity, and improved diabetes care [25, 30]. Peer support persons encourage and motivate individuals with diabetes, using their own personal disease experience to help others learn how to manage their diabetes and motivate action towards improved diabetes care [31, 32]. This practical approach to addressing disease self-management including treatment adherence may work in underserved communities [32]. However, to our knowledge, this is the first study to engage African Americans with type 2 diabetes who are successfully taking their medicines as peer support persons for African Americans with diabetes who are having challenges with taking medicines [33, 34]. As peer supporters, they may be able to enhance patient activation skills including expressing concerns with providers, and asking questions during clinic/pharmacy visits, leading to increased engagement in self-management [25, 35]. Because African Americans are likely to have mistrust of the healthcare system, providing culturally appropriate peer support can enhance treatment adherence in ways that cannot be accomplished by healthcare providers through clinic visits [36].

Peers Supporting Health Literacy, Self-Efficacy, Self-Advocacy, and Adherence is a theory-driven educational-behavioral intervention that provides African Americans with (1) culturally tailored diabetes and medication information, (2) one-on-one peer support from African Americans with diabetes, and (3) self-efficacy/provider communication skills to improve medication adherence. In this intervention, African Americans with type 2 diabetes, who were adherent to their diabetes medicines (Ambassadors), were matched with African Americans with type 2 diabetes who were nonadherent to diabetes medicines (Buddies). Intervention components included group education and phone follow-ups to address psychosocial factors including reducing misperceptions/misinformation about diabetes and medicines, building self-efficacy and patient-provider relationships, and providing support for diabetes management by sharing/modeling successful diabetes self-management strategies. The study objective was to evaluate the feasibility and acceptability of this peer-supported diabetes medication adherence intervention for African Americans.

### Conceptual framework

Behavior change within the intervention is conceptualized using the Information-Motivation-Behavioral skills model focusing on specific constructs needed for successful diabetes self-management including *information* “an initial prerequisite for enacting a health behavior”, *motivation*—taking into account beliefs about the intervention and attitudes toward adherence and social support for engaging in a behavior [37] and *behavioral skills*—increasing self-efficacy and activation [38–40]. The self-regulatory model was integrated with the information/beliefs component of the Information-Motivation-Behavioral skills model as it addresses misinformation/beliefs about illness and medicines by educating on the concerns about medicines and providing information on the necessity of medicines [41–44].

## Methods

### Study aims

The pilot was conducted to assess the feasibility and acceptability of the educational-behavioral intervention. As well, we explored if there was a signal of effect in exploratory outcomes, change in blood glucose, medication adherence, beliefs about diabetes and diabetes medications, self-efficacy for taking medications, health literacy, social support, and patient-provider communication.

### Design

This feasibility study used a single group, pre-post intervention study design with African American buddies (*n*=22) with type 2 diabetes [45].

### Participants

There were twenty-two buddies who were program participants, with seventeen ambassadors who provided peer support to the buddies. Ambassadors and buddies had to meet the following inclusion criteria: aged 30–65 years old, diagnosed with type 2 diabetes, self-identified as Black/African American, able to speak/read English, self-reported being prescribed at least one oral diabetes medication, and had access to and could use a cellular phone during the study period. Additional criteria included ambassadors self-reporting as being adherent to diabetes medicines (score of 11 on the Adherence to Refills and Medications-Diabetes Scale), while buddies self-reported as being nonadherent (score of greater than 11 on the Adherence to Refills and Medications-Diabetes (ARMS-D) Scale) [48]. The exclusion criteria was not taking oral diabetes medicines. Ambassadors were matched to buddies based on gender in a 1:1 or 1:2 pair.

### Procedures/methodology

#### Recruitment

Purposive sampling was used for recruitment. To recruit ambassadors, we worked with our local community stakeholders, as well as invited ambassadors from our prior study to help identify potential ambassadors [47, 48]. Prior ambassadors were also asked to consider being an ambassador again. Buddies were recruited from churches, apartments, food pantries, diabetes support groups, and senior centers using IRB-approved passive and active recruitment approaches such as posting flyers in the community locations and directly meeting with potential participants face to face (prior to COVID-19) based on referrals from the community partners. As well, we used radio advertisements. Based on word of mouth from community members, potential participants could also call the project coordinator’s phone. Each participant was screened either on the phone or face to face to assess their eligibility for the inclusion criteria. Participants received a total of $130 for completing the intervention and data collection for all outcomes.

#### Training of ambassadors

Each ambassador completed a 4-h training led by the PI and the Wisconsin Network for Research Support to prepare them for their role as peer supporters including talking to their buddies during the group sessions and phone calls. Role playing activities between ambassadors was used as the approach for training.

#### Setting

The intervention was conducted in two Mid-western cities. The first city has about 200,000 people, while the second location was a city of about 600,000 people.

#### Intervention

Supporting Health Literacy, Self-efficacy, Self-Advocacy, and Adherence was an 8-week culturally tailored peer-based educational-behavioral intervention that consisted of group education and individual phone-based follow-up support with ambassadors (Table 1).

**Table 1:** Details of a 8-week intervention

Week	Format	Description
**1**	Group education	A diabetes educator targets negative illness beliefs with a focus on the cause and consequence of diabetes.
**2**	Group education	A pharmacist reframes medication beliefs to decrease medication concerns and increase necessity in medicines.
**3**	Group education	A physician discusses hemoglobin A1C and blood glucose.
**4**	Phone	Discuss self-efficacy, positive living, and coping with diabetes.
**5**	Phone	Provide support for addressing fear, frustration, and emotional distress.
**6**	Phone	Discuss self-advocacy in provider communication/relationship building.
**7**	Phone	Discuss family/community bonding and maintaining cultural experiences.
**8**	Phone	Set a goal for improving health.

##### Weekly group education sessions (weeks 1–3)

The first 3 weeks of the intervention were conducted as face-to-face group sessions prior to COVID-19 and then switched to virtual sessions due to pandemic restrictions. These weekly 2-h group education sessions were led separately by a physician, pharmacist, and diabetes educator. For each group session, there was a presentation by the health care professional leading the session, small group activities for ambassadors and buddies to interact and discuss questions and presentation content. The virtual format of the group session was shortened to 90 min, and only included the presentation by the health professional and time to ask/discuss questions. It was important for the ambassador to attend each group session with their buddy so that they could learn about the intervention content together and build social trust to support interactions.

##### Weekly phone calls between ambassadors and buddies (weeks 4–8)

Five weekly phone calls were completed with buddies after the completion of the group education sessions. Each phone call from the ambassador was guided by a standardized phone call manual with detailed guidance, and a large at-a-glance call guide summarizing the detailed guidance, which were developed by the PI and the Wisconsin Network for Research Support [47]. During the phone calls, ambassadors provided peer support to buddies, answered questions and concerns about diabetes, responded to buddy needs, and delivered the standardized intervention content. Ambassadors talked to buddies for 15–30 min weekly (Tables 1).

### Intervention fidelity

To evaluate the fidelity of the intervention—the extent to which the intervention is delivered as it was intended—we audio-recorded the group sessions to determine how the intervention was implemented [49]. Also, project coordinators completed weekly phone calls to ambassadors and buddies to document the intervention content discussed during the phone call.

Approval from the Institutional Review Board of a Mid-Western university was obtained for all research activities (STUDY ID: 2019-0721). All participants provided written consent to participate. The study was registered at https://clinicaltrials.gov/ct2/show/NCT04028076.

### Data sources

#### Recruitment, retention, and adherence

To assess the feasibility and acceptability of the trial, we examined (1) recruitment, (2) retention rates, (3) intervention adherence, and (4) buddies’ reactions to the intervention including their perceived impact on medication adherence. We aimed for a recruitment rate of 80% for both ambassadors and buddies and measured it by the number of ambassadors and buddies we enrolled as a proportion of our initial enrollment targets.

We defined the participant retention rate as the number of buddies that completed an HbA1c test as an outcome assessment at a 3-month follow-up compared to the number at baseline, with a goal for 80% of participants completing baseline assessments.

To assess the feasibility of gathering outcome data and assess for a signal of effect, buddies completed self-administered 20-min surveys and hemoglobin A1c (HbA1c) tests assessing changes in outcomes. All baseline pre-intervention data including surveys and clinical outcomes were collected in the first week, and follow-up data was collected 3 months post-intervention. A nurse/nursing assistant study team member completed HbA1c and blood pressure measurement as clinical indicators of medication adherence.

Intervention adherence was measured by recording attendance at group sessions and the number of five required phone calls ambassadors completed. A call was considered completed if they spoke for 15 min or longer and addressed one or more of the intervention topics. The study team conducted weekly check-ins with each ambassador to record the approximate length of the call and intervention topics discussed.

### Outcome measures

We examined the effect of the intervention in changing outcomes using a survey, including validated measures such as self-reported medication adherence, assessed by the Adherence to Refills and Medications-Diabetes Scale, a measure with a score range of 11–44, where the higher score indicates better adherence to taking or refilling diabetes medications, hemoglobin A1c, illness beliefs (beliefs about diabetes)—the Brief Illness Perception Questionnaire, a 10-item scale where higher scores represent stronger illness perceptions, medication beliefs (beliefs about diabetes medicines)—Beliefs about Medicines Questionnaire, a 10-item questionnaire with two sub-scales, necessity beliefs, and concern beliefs and a score range of 5–25 for each subscale, the higher score means stronger concern beliefs or necessity beliefs about the medicine, medication self-efficacy—Self-Efficacy for Adherence to Medication Scale, a 13-item questionnaire with a score range of 13–39, where the higher score represents greater self-efficacy on medication use, diabetes related psychosocial self-efficacy—the Diabetes Empowerment Scale–Short Form, and 8-item scale with a range of scores from 8 to 40, with higher scores indicating higher patient self-efficacy health literacy—Newest Vital Sign, a 6-item questionnaire with a score range 0–6, where a higher score represents a higher health literacy level for the participant, social support—Social Support section of Diabetes Care Profile, and patient-provider communication—Patient’s Perceived Involvement in Care Scale, a 13-item questionnaire with a score range of 0–13, where the higher score stands for better communication between patients and providers [46, 50–56] (Table 2). Socio-demographic data collected included age, gender, race/ethnicity, and clinical characteristics—self-reported health and number of medications used.

**Table 2:** Quantitative outcome measures

Feasibility outcomes	Measures
Ambassador and buddy recruitment	Recruitment statistics
Ambassador attrition	Completion of a 8-week intervention
Buddy retention	Primary outcome assessment data at a 3-month follow-up
Ambassador participation in training and intervention sessions	Meeting/training attendance records
Buddy participation in intervention sessions	Meeting attendance records
Satisfaction with group sessions	Post session evaluation
**Intervention outcomes**	**Measures**
*Primary*	
Blood glucose	Hemoglobin A1c (Hba1c)
Medication adherence	Adherence to Refills and Medications Scale- Diabetes (Mayberry et al., 2013)
*Secondary*	
Blood pressure	Systolic and diastolic blood pressure
Diabetes health beliefs	Brief Illness Perception Questionnaire (Broadbent, Petrie, Main, & Weinman, 2006)
Beliefs about diabetes medicines	Beliefs about Medicines Questionnaire (Horne, Weinman, & Hankins, 1999)
Diabetes medication self-efficacy	Self-Efficacy for Adherence to Medication Scale (Risser, Jacobson, & Kripalani, 2007)
Diabetes psychosocial self-efficacy	Diabetes Empowerment Scale – Short Form (Anderson, Funnell, Fitzgerald, & Marrero, 2000)
Health literacy	Newest Vital Sign (Weiss et al., 2005)
Patient provider communication	Patient’s Perceived Involvement in Care Scale (Lerman et al., 1990)
Social support	Social Support section of Diabetes Care Profile (Fitzgerald et al., 1998)

### Sample size justification

Since this was a feasibility pilot study, there was no power analysis for sample size determination. However, the rationale for the sample size was based on prior studies that show that there should be 10 to 12 individual per group for a pilot feasibility study [57–59]. Since we did not have a control group in this trial and this was a single group pre-post design, we aimed for this required sample size.

One aspect of acceptability, satisfaction with group education sessions, was evaluated through brief 7-item questionnaires administered immediately following each group education session. As well, semi-structured 60-min face-to-face or virtual interviews (due to COVID-19 restrictions) were scheduled with all buddies at the conclusion of the 8-week intervention to assess for intervention acceptability, based on their experience. All interviews were audio recorded and conducted by three trained research assistants with experience conducting qualitative interviews. The interviews were then transcribed verbatim by a certified transcriptionist. Sample questions are provided in Table 3. The interviews allowed us to explore details about buddies’ view of the intervention, its impact, and process [60].

**Table 3:** Sample interview questions

How easy or hard was it finding a time to talk with your ambassador?	
How comfortable did you feel talking to your ambassador?	
How helpful was it talking to a peer ambassador over the phone?	
Are there different ways you would have liked the calls structured?	
If you could change the intervention, what would you change?	
What part of the intervention was of most and least benefit to you?	

### Data analysis

Descriptive statistics were used to summarize the feasibility outcomes including the recruitment, retention, and intervention adherence rates. Similarly, descriptives including the mean and standard deviation (SD) were used to summarize the socio-demographic and clinical characteristics of the study sample. Using baseline and 3-month follow-up outcomes, we calculated percent change for HbA1c, self-reported medication adherence, and all other psychosocial measures (e.g., illness beliefs, social support). Given the non-normal distribution and small sample size, nonparametric Wilcoxon signed-rank tests were conducted to assess for differences in mean scores of outcome variables at baseline compared with a 3-month follow-up. Effect size was then calculated using the Wilcoxon signed-rank test statistics (SPSS Statistics Version 26). All statistical analyses were carried out using SPSS version 26.

Acceptability was assessed through post-intervention qualitative interviews with each buddy eliciting their perceptions of each component of the intervention. We conducted qualitative content analysis using an inductive open coding approach, comparing themes across buddies’ individual interview responses. NVivo 12 (QSR International Melbourne) was used to organize and categorize the themes for the analysis. This process included, initially reading the transcripts to achieve immersion; reading the data line by line; creating a priori codes based on the interview guide questions (e.g., reactions to group sessions, reactions to peer support from ambassador, reactions to phone calls with ambassadors); and developing and organizing the themes [61]. Analysis occurred until data saturation, i.e., we could not find new dimensions within the data [62–64]. Four research assistants, all of whom were skilled in qualitative research coded the transcripts independently. The research assistants then met with the PI and as a group, we discussed the similarities and divergences before reaching agreement on all final themes. To ensure the rigor of the qualitative data, which allows us to verify the findings throughout the data analysis process, we focused on analytic criteria such as credibility, conformability, and transferability. Credibility refers to the faithful interpretation of participant views. To increase credibility, we used investigator triangulation (i.e., multiple coders involved in the data analysis) and member checking—a synthesis of the findings/result summary mailed to participants to ensure that the descriptions are salient and credible. All participants noted no change was needed to the themes [65]. As well, to increase confirmability, i.e., the objectivity, or potential congruence between qualitative researchers, we resolved all discrepancies in three separate discussions involving the PI and research assistants who coded the data. Prior, the independent coders had met to discuss identified NVivo comparisons of codes and interpretations. Lastly, transferability refers to the potential for extrapolation to other groups. In our approach, transferability was increased by interviewing people from different cities where the intervention was completed.

## Results

### Demographic and clinical characteristics of buddies and ambassadors

Buddies (Table 4) and ambassadors (Table 5) were similar in age, had been diagnosed with diabetes for similar lengths of time—a mean of approximately 10 years, and were mostly female. The mean number of diabetes medications the buddies reported taking was 1.67 (± 0.8).

**Table 4:** Demographics and clinical characteristics of buddies (*n*=21)^a^

Variables	
Age (mean, standard deviation)	56 (8.7)
Gender, *n* (%)	
Female	13 (62)
Education, *n* (%)	
Some high school	2 (9.5)
High school graduate or GED	5 (23.8)
Trade school	1 (4.8)
Some college	4 (19.0)
Associate degree or a 2-year college degree	2 (9.5)
Bachelor’s degree or a 4-year college degree	3 (14.3)
Master’s degree	2 (9.5)
Advanced degree (e.g., J.D., M.D., PhD)	1 (4.8)
Annual household income, *n* (%)	
Less than 20,000	13(61.9)
Equal or more than 20,000	7 (33.3)
Number of chronic illnesses (mean, standard deviation)	2.4 (1.4)
Number of diabetes medications (mean, standard deviation)	1.7 (0.8)
Years of diabetes diagnosed (mean, standard deviation)	10 (8.9)
Health status, *n* (%)	
Poor	1 (4.80)
Fair	15 (71.4)
Good	3 (14.3)
Excellent	1 (4.8)

^a^Missing value

**Table 5:** Demographics and clinical characteristics of ambassadors (*n*=15)^a^

Variables	
Age (mean, standard deviation)	57 (7.5)
Gender, *n* (%)	
Female	8 (53.3)
Years of diabetes diagnosed (mean, standard deviation)	10 (7.5)

^a^Missing value

#### Feasibility outcomes

##### Recruitment

Twenty-two African Americans with diabetes (buddies) enrolled and were paired with 17 African American ambassadors. Given the recruitment goal of enrolling 30 buddies, this represents a recruitment rate of 22/30 (73%). The enrollment goal for ambassadors was 20, for a recruitment rate of 17/20 (85%). We set a feasible recruitment rate of 80%, so, we were not able to reach our recruitment goal for the buddies.

##### *Retention*

One buddy was lost to follow-up and 21 buddies completed assessment for the primary exploratory outcomes HbA1c and medication adherence at all three data collection times, representing a 95% retention rate. One ambassador was lost to follow-up, and a second ambassador could not meet the program responsibilities. Ambassadors completed the program at a 94% retention rate. We exceeded our retention for buddies and ambassadors based on our anticipated retention rate of 80%.

##### Intervention adherence

Both buddies and ambassadors had excellent intervention adherence, with buddies having a mean attendance of 7.76 out of 8 sessions/phone calls and ambassadors completing > 99% of the 105 intervention calls with buddies.

##### Reactions to intervention components

Twenty-one semi-structured interviews were completed to elicit buddy’s feedback on the intervention.

### Acceptability of the intervention

Overall, buddies reported that they found the components of the intervention and the procedures to be acceptable. Buddies greatly enjoyed the group education sessions:

Well, the aspect of the program, for me, the most benefit was coming to the group sessions. I like that a lot…and I was able to…get direct information from a pharmacist and a doctor and a diabetes educator. (Buddy 5)

Quantitative data collected to assess participant satisfaction with the group sessions demonstrated that across all three separate sessions, more than 75% of buddies reported that they liked the session and liked debriefing the sessions with their ambassador “very much.” More than 90% of buddies responded that the material was presented clearly, and they had sufficient time to make comments and answer questions.

Buddies reported that the one-on-one peer support phone calls with ambassadors were very enjoyable:

You know, I liked the phone calls, the Ambassador phone call. You know, I, at first, I was skeptical about talking with someone on the phone, because I didn’t know that person. But once I allowed myself to answer the phone to talk to her, it was good. (Buddy 9)

#### Perceived benefits of the intervention

Themes suggest that the buddies found the intervention beneficial in several ways:

Buddies valued ambassador’s validation and encouragement of positive self-management behaviors, reported improved knowledge of diabetes, increased perception of the necessity of diabetes medicines, and improved provider communication.

Participants reported feeling validated and encouraged through their discussions with their ambassador.

Through this program, I’m learning that I do have a voice and what I’m going through matters. It matters, and someone wants to know what we’re going through to kind of, you know, make things better for us. (Buddy 3)

Participants mentioned having improved knowledge of diabetes.

It [Peers LEAD] helped me better understand my disease in terms of how to take care, better care of myself, what to eat, what not to eat, and making good choices about the things I should put in my body, you know, that affects my diabetes. (Buddy 9)

Buddies also commented on how speaking with the ambassador seemed to have improved their communication with providers;

I learned to not be running from my doctor’s office... not to be so scared to go to the doctor and talk to you doctor and ask questions… They ain’t the enemy. They just to support and help you.” (Buddy 21)

Overall, buddies appreciated the social support received from the ambassador.

It [support from Peer Ambassador] meant a lot to me. Like I said, the first thing to know that I'm not alone, and the second thing, we can exchange helpful hints with each other on how to control our diabetes and have a real, a happy life with that diabetes. (Buddy 7)

Table 6 provides other representative quotes related to themes including changing beliefs about diabetes and improved provider and pharmacist communication. Several buddies expressed that the intervention would be valuable for other African Americans with diabetes and recommended that the intervention be expanded. This speaks to their perceptions of the acceptability and feasibility of the intervention for the larger African American community.

So for me, it [Peers LEAD] was a big help, and I think for others, it will be a great help, because it’s, and having that Peer Ambassador to talk to would be a great help, a doctor coming and telling you that you don’t have to be up to six, like a seven, in the middle of seven or a little bit lower, you know…So I think all that would be very important to people especially who have not taken the time to read up on it or no one else has sat down and tried to educate them about what they have. (Buddy 8

**Table 6:** Buddies perceptions of intervention feasibility and acceptability

Theme	Quotes
Acceptability	
Overall acceptability of peers LEAD	
Liked the group education sessions	“Well, the aspect of the program, for me, the most benefit was coming to the group sessions. I like that a lot…and I was able to…get direct information from a pharmacist and a doctor and a diabetes educator.” (Buddy 5) “This group [education session] was very helpful, you know, because for one thing, you're interacting with people that, in the field of profession, of being diabetic, you know. They help us. They taught us what we can do, give us suggestions . . . and at the same token, you know, they like the feedback…” (Buddy 6) “You know, I thought the three [group education sessions] meetings were pretty decent. The doctor one, by far, was the most informative and interesting. The pharmacist, pharmacy one was okay...I guess the doctor’s meeting [group education session] where she talked. I guess that information was very, was probably the most beneficial to me.” (Buddy 19) “I liked the, I really liked the doctor input, and then I liked the educational part, because without education, you can’t proceed and get better. So I liked that really. The education is the key. Because if you educate a person, then they’ll be able to do it even if the doctor or the pharmacist is not around.” (Buddy 24)
Phone calls with ambassadors was helpful	“And then they [Ambassador] told me they was going by their script too, so I thought the script was pretty good. Whatever they got taught, they, I was impressed that they was following what you all had taught them to the letter. And I enjoyed that, you know…Y’all gave them [Ambassadors] stuff to touch on with people [Buddies] and stuff like that…I think that helped a lot that I knew they was trained to do it, right.” (Buddy 2) “You know, I liked the phone calls, the Ambassador phone call. You know, I, at first, I was skeptical about talking with someone on the phone, because I didn’t know that person. But once I allowed myself to answer the phone to talk to her, it was good.” (Buddy 9) “They [phone calls] were just pretty helpful. How sometimes, some of them days…I forgot to take my medicine, or, you know, we talked about stuff I needed to do and just different stuff.” (Buddy 23)
Benefits gained from the intervention	
Satisfaction with ambassadors	“It's [talking with Ambassador] helpful to me because it's someone that shares something that I’m going through. And I always felt like, you know, it was just me against everything. Nobody else knew what I was going through. Nobody else could relate…But with the peer ambassador understanding that, she has shared what, you know, the same thing, that I'm able to relate to it more where I could say, okay, that makes sense, you know. So just talking with her, period, just made me feel like somebody understands me, and they know what I'm going through, as opposed to my regular primary doctor or a family member.” (Buddy 3) “It was, because how can I say, she [Ambassador] was there when I needed somebody to be there. I mean, yeah, I spoke with my doctor, but it’s good sometime to have that sounding board…it was good to be able to speak to her [Ambassador]. Again, she’s [Ambassador] had metformin, she knows about metformin, she’s got diabetes. So there’s somebody I could talk to that I could relate to…So… that was great, yeah. I was glad to have her there.” (Buddy 8) “Oh, he [PA] was wonderful. I mean, you know, like I said, we shared a lot. And I liked the idea that he wasn’t bougie or he didn’t seem like he knew everything. He goes, do this, do that, you know. I mean, he was open to suggestions for himself, you know. And he was open to suggestions that had something to do with me. And I was open to them, I mean, we spoke one to another as if we have known each other all our lives.” (Buddy 25)
Changed or reinforced beliefs about diabetes	“I learned that the stereotype that only black people have it [diabetes] was not true and that it’s not a curse.” (Buddy 1)
	“There [in Peers LEAD] was a lot of dispelling of the myths and what people thought diabetes was and what that meant. And that made me more confident. That made me more receptive to…taking my insulin and taking my medication because at first, it was just like, no, no, no, no, no… I'm still working on consistency, but I'm more consistent than I was than when I first started the program.” (Buddy 15)
Increasing awareness and self-efficacy related to communicating with providers	“Since I started the study [Peers LEAD] … because I got more information about talking with like people like [Provider presenter] and the professor and everybody…I think I got a better handle on talking to the doctor now…more confidence.” (Buddy 2)
	And then work with my doctor more closely to discuss my needs, that's a goal that I have.…And I need to communicate more effectively with my doctor about all my health issues. (Buddy 5)
Improved communication with provider/pharmacist since program	**“**I think I can communicate with them [provider / pharmacist] better since…did the study [Peers LEAD], because I got a little bit more insight on what the questions to ask.” (Buddy 2)
	“When my numbers had started going down, I actually picked up the phone and called my doctor and just let her know the, my numbers had went down, and I was feeling great and doing great. So it [Peers LEAD] kind of encouraged me to give that push to actually call my doctor.” (Buddy 21)
Acceptability of structure and process of intervention	“Only thing I can say, I can give you all five stars. I thought it was five-star treatment. And that’s how, then you got the partnership room, and then, and the space room. So I think y’all have had a five-star layout. I enjoyed that. I thought y’all did good, and y’all had stuff to eat. So I think y’all went out of y’all way to, with the program. I think y’all did great. I thought it was great planning.” (Buddy 2)
Feasibility	
Views on intervention for larger population *Great need of the program for other African Americans* *Program is essential because there are many who lack knowledge of managing diabetes*	“It's a great program [Peers LEAD], and I really hope you all continue it and expand it, because we have a whole lot of people out there that's diabetic, black African-American people that's diabetic and don't know anything about diabetes… they can expand it and continue to draw them in where they can learn more about diabetes and take control of their life with diabetes.” (Buddy 7) “…it [Peers LEAD] was very helpful, and I think it will help, going forward, I think it will help a lot of people that really don’t know. I didn’t know.” (Buddy 8) “So, and, like I said, I think it [Peers LEAD] would be great, so please don’t stop, because there’s a lot of people out there like me that don’t know, and they’re focused on one or two things, but there's a whole big picture that they need to see. So please don’t stop.” (Buddy 8) It’s [Peers LEAD] very educational. Yeah. Very education. Personally, I think they should keep going because there are a lot of people that’s out there that suffer with this disease and don’t know how to, either they don’t know how to deal with it, or they don’t want to deal with it. And a lot of people need to be, you know, reached out at because it’s been around for a long time, you know. (Buddy 25 “So, yes, and I think more people, if they had a chance to go through this [Peers LEAD], they would appreciate …the whole thing, because, I mean, you’ve got a pharmacist, you’ve got a doctor coming in…you got somebody that, an ambassador that can relate to you, not just somebody that’s never had diabetes talking to you and prescribing medications…So I think this, I think the whole program is very informative and would be helpful to those who are on metformin, who have diabetes type 2…” (Buddy 8)
Barriers to the intervention	
Feedback on challenges with intervention—recommendations for things they might change	Survey was very long; felt rushed filling it out Have ambassador help filling it out Food without labels—difficult for diabetes More opportunities to mingle and get to know one another/more interaction with other buddies and ambassadors outside of program (e.g., social gatherings) Would have liked to hear more about other buddies’ life stories More time for education presenters to ask questions Handouts to take home from (physician) presentation Provide evaluation form at beginning of session so they know what to be thinking about

Buddies also reported some aspects of the intervention they would like to see changed and/or improved. These included (1) streamlining the questionnaire, allowing participants more time to complete the questionnaire and offering participants help with its completion; (2) allowing the group education presenters more time, especially to respond to participant’s questions; and (3) building in more opportunities during the group education for buddies and ambassadors to interact and get to know one another.

#### Clinically significant signal

There were no statistically significant differences in mean scores at the 3-month follow-up compared to baseline for HbA1c and self-reported medication adherence. However, the percent change in mean outcomes reflects improvements in HbA1c and medication adherence and reductions in buddies’ negative beliefs about diabetes and an increase in their beliefs about the necessity of diabetes medicines (Table 7).

**Table 7:** Baseline to 3-month changes in clinical and psychosocial outcomes (*n*=20)

Outcome	Mean (SD)		Percent change	Effect size
	Pre	Post		
**Primary outcomes**				
Hemoglobin A1c^a^	8.0 (2.4)	7.8 (2.1)	−3%	0.14
Medication nonadherence	16.1 (4.0)	14.7 (3.9)	−9%	0.35
**Secondary outcomes**				
Blood pressure, systolic^a^	127.6 (19.9)	129.9 (20.0)	+2%	0.20
Blood pressure, diastolic^a^	80.9 (13.7)	81.9 (10.2)	+1%	0.13
Necessity of taking medicines	19.1 (5.7)	20.3 (5.0)	+7%	0.30
Concerns about taking medicines	16.6 (5.6)	15.9 (5.2)	−4%	0.20
Negative beliefs about diabetes	40.3 (11.6)	36.7 (10.6)	−9%	0.38
Self-efficacy	31.9 (5.0)	33.0 (5.2)	+4%	0.20
Patient provider communication	15.6 (2.0)	15.9 (2.2)	+2%	0.11
Social support	59.2 (22.6)	60.1 (17.2)	+2%	0.16
Diabetes empowerment	30.9 (10.3)	31.3 (7.6)	+1%	0.07
Health literacy	3.1 (1.9)	2.9 (2.1)	−3%	0.08

^a^Baseline data for clinical outcomes was collected for a buddy who did not complete the psychosocial measures, so *n*=21 for these outcomes

## Discussion

In this pilot study, we assessed the feasibility and acceptability of a culturally appropriate peer-supported educational-behavioral medication adherence intervention for African Americans with type 2 diabetes. We used a culturally tailored combination of group education and one-on-one peer support from African Americans with medication adherence to address the specific psychosocial (e.g., self-efficacy and culturally specific beliefs about diabetes), social (e.g., peer support), and other factors (e.g., lack of access to information, mistrust of providers) identified from our prior work with African Americans with type 2 diabetes to influence medication adherence [33, 34, 47].

Results showed a recruitment rate of 73% which was lower than expected based on our a priori 80% rate. However, our recruitment was impacted due to COVID-19. We had to use radio advertisements as our only method of recruitment instead of working directly with community partners. Also, some individuals were not interested in a virtual format. Despite this recruitment challenge, we had success with retention at a rate of ~95% of buddies and ambassadors completing the 8-week intervention and providing data for the data collection time points.

Since this pilot was not powered to detect statistically significant effects, we did not expect to find quantitative results showing statistically significant outcomes. Our feasibility study aimed to see a signal of change in HbA1c and medication adherence, which was detected in these exploratory outcomes as well as beliefs in medicines, beliefs about diabetes, self-efficacy, social support, and patient-provider communication. A future fully powered pilot randomized controlled efficacy trial is needed to show statistically significant improvements in HbA1c as an indicator reflective of improved medication adherence over time, as well as compare it with usual care.

Nevertheless, the qualitative results support the quantitative results in showing that buddies benefited from the intervention. For example, the in-depth qualitative interviews with buddies revealed the acceptability of the intervention to buddies including overall perceived benefit of the intervention because of the knowledge gained about diabetes, the addressing of misbeliefs about diabetes and diabetes medicines, and the building of self-efficacy to communicate with providers. Buddies appreciated the value of peer support from a peer ambassador, someone who was also African American and managing their diabetes well.

The self-reported reduction in medication nonadherence is supported by prior literature where the utilization of peer support models in disease self-management interventions for African Americans is shown to be effective [26, 29]. This study confirms prior knowledge suggesting that peer support provides encouragement and motivation for diabetes self-management and may significantly improve medication adherence for African Americans. Additionally, peer support and group education sessions with ambassadors may have helped to change the patient’s self-efficacy and confidence in speaking with their providers about their illness and medicines and possibly enhance communication with providers.

Self-efficacy to communicate with providers and addressing illness and medicine misperceptions are crucial in creating effective diabetes self-management interventions for African Americans. As shown in this study, poor medication adherence and poorer outcomes for African Americans may be in part due to culturally influenced health beliefs that do not align with current care. Implications for clinical practice includes achieving care for African Americans that acknowledges and addresses cultural differences between patients and provider perceptions of illness and medicines [18, 66, 67].

Buddies reported high satisfaction with the facilitators of the intervention group sessions, including being satisfied with the structure and process of the intervention such as the one-on-one weekly phone calls with ambassadors. Provider and pharmacist-led group sessions with reinforcing peer support from ambassadors seemed like a feasible approach to addressing buddies’ beliefs and misinformation about diabetes and medicines, increasing awareness of pharmacists and providers as resources for medication adherence information, and building buddies’ skills and self-efficacy related to communicating with healthcare professionals.

There were some study limitations. Recruiting enough African Americans was challenging. Though the study team had developed strong partnerships with several community-based organizations in each of the study locations, there were recruitment limitations due to the COVID-19 pandemic occurring in the middle of the study implementation, leading to further restrictions during the study. As well, there were retention issues, due to the switch from face-to-face to a virtual form of delivering the intervention. Despite the challenges in retaining program participants, we sustained our moderate retention rates using weekly check-in phone calls with each ambassador and buddy. These calls served two purposes including to assess for intervention fidelity and to offer support to the ambassador and buddy in the navigation of peer support. Though we showed a change in the exploratory outcomes of medication adherence and HbA1c, the results should be interpreted with caution as we had a small sample and no control group.

## Conclusion

This peer-supported educational-behavioral intervention to address beliefs about diabetes and diabetes medicines, and self-efficacy related to medication adherence among African Americans appeared acceptable. African Americans with type 2 diabetes may benefit from group education and one-on-one weekly support from an African American peer with type 2 diabetes who is managing their diabetes medicines well. They have the potential to provide peer support and enhance medication adherence. The data provide support for a future randomized controlled trial to test the efficacy of the intervention, as well as examine the possibility of its integration into other existing evidence-based diabetes self-management programs.

## Data Availability

When requested.

## Abbreviations

HbA1c: Hemoglobin A1c
COVID-19: Coronavirus

## References

[1] Marshall M (2005). Diabetes in African Americans. Postgrad Med J.

[2] Gu K, Cowie CC, Harris MI (1998). Mortality in adults with and without diabetes in a national cohort of the U.S. population, 1971–1993. Diabetes Care.

[3] Delamater AM (2006). Improving patient adherence. Clin Diabetes.

[4] Shenolikar RA, Balkrishnan R, Camacho FT, Whitmire JT, Anderson RT (2006). Race and medication adherence in Medicaid enrollees with type-2 diabetes. J Natl Med Assoc.

[5] Krapek K, King K, Warren SS, George KG, Caputo DA, Mihelich K (2004). Medication adherence and associated hemoglobin A1c in type 2 diabetes. Ann Pharmacother.

[6] Hu D, Juarez DT, Yeboah M, Castillo TP (2014). Interventions to increase medication adherence in African-American and Latino populations: a literature review. Hawai'i J Med Public Health.

[7] McQuaid EL, Landier W (2018). Cultural issues in medication adherence: disparities and directions. J Gen Intern Med.

[8] Schectman JM, Nadkarni MM, Voss JD (2002). The association between diabetes metabolic control and drug adherence in an indigent population. Diabetes Care.

[9] Thomas K, Nilsson E, Festin K, Henriksson P, Lowén M, Löf M (2020). Associations of psychosocial factors with multiple health behaviors: a population-based study of middle-aged men and women. Int J Environ Res Public Health.

[10] Lewis LM (2012). Factors associated with medication adherence in hypertensive blacks: a review of the literature. J Cardiovasc Nurs.

[11] Lewis LM, Askie P, Randleman S, Shelton-Dunston B (2010). Medication adherence beliefs of community-dwelling hypertensive African Americans. J Cardiovasc Nurs.

[12] Rao D, Maurer M, Meyer J, Zhang J, Shiyanbola OO (2020). Medication adherence changes in Blacks with diabetes: a mixed methods study. Am J Health Behav.

[13] Dimatteo MR, Giordani PJ, Lepper HS, Croghan TW (2002). Patient adherence and medical treatment outcomes: a meta-analysis. Med Care.

[14] Buckley L, Labonville S, Barr J (2016). A systematic review of beliefs about hypertension and its treatment among African Americans. Curr Hypertens Rep.

[15] Petrie KJ, Perry K, Broadbent E, Weinman J (2012). A text message programme designed to modify patients' illness and treatment beliefs improves self-reported adherence to asthma preventer medication. Br J Health Psychol.

[16] Keogh KM, White P, Smith SM, McGilloway S, O'Dowd T, Gibney J (2007). Changing illness perceptions in patients with poorly controlled type 2 diabetes, a randomised controlled trial of a family-based intervention: protocol and pilot study. BMC Fam Pract.

[17] Mann DM, Ponieman D, Leventhal H, Halm EA (2009). Predictors of adherence to diabetes medications: the role of disease and medication beliefs. J Behav Med.

[18] Shiyanbola OO, Ward E, Brown C (2018). Sociocultural influences on African Americans’ representations of type 2 diabetes: a qualitative study. Ethn Dis.

[19] Rhyant B, Skinner CS, Krier C, Willis DR, Ballard D, Smith-Howell E (2012). Computer-delivered tailored intervention improves colon cancer screening knowledge and health beliefs of African-Americans. Health Educ Res.

[20] Martin MA, Catrambone CD, Kee RA, Evans AT, Sharp LK, Lyttle C (2009). Improving asthma self-efficacy: developing and testing a pilot community-based asthma intervention for African American adults. J Allergy Clin Immunol.

[21] Henderson J, Wilson C, Roberts L, Munt R, Crotty M (2014). Social barriers to type 2 diabetes self-management: the role of capital. Nurs Inq.

[22] Colleran KM, Starr B, Burge MR (2003). Putting diabetes to the test: analyzing glycemic control based on patients’ diabetes knowledge. Diabetes Care.

[23] McPherson ML, Smith SW, Powers A, Zuckerman IH (2008). Association between diabetes patients' knowledge about medications and their blood glucose control. Res Social Adm Pharm.

[24] Heisler M, Faul JD, Hayward RA, Langa KM, Blaum C, Weir D (2007). Mechanisms for racial and ethnic disparities in glycemic control in middle-aged and older Americans in the health and retirement study. Arch Intern Med.

[25] Heisler M (2007). Overview of peer support models to improve diabetes self-management and clinical outcomes. Diabetes Spectr.

[26] Long JA, Jahnle EC, Richardson DM, Loewenstein G, Volpp KG (2012). Peer mentoring and financial incentives to improve glucose control in African American veterans: a randomized trial. Ann Intern Med.

[27] Heisler M, Piette JD (2005). "I help you, and you help me": facilitated telephone peer support among patients with diabetes. Diabetes Educ.

[28] Heisler M, Vijan S, Makki F, Piette JD (2010). Diabetes control with reciprocal peer support versus nurse care management: a randomized trial. Ann Intern Med.

[29] Heisler M, Choi H, Mase R, Long JA, Reeves PJ (2019). Effectiveness of technologically enhanced peer support in improving glycemic management among predominantly African American, low-income adults with diabetes. Diabetes Educ.

[30] Funnell MM, Anderson RM (2004). Empowerment and self-management of diabetes. Clin Diabetes.

[31] Qi L, Liu Q, Qi X, Wu N, Tang W, Xiong H (2015). Effectiveness of peer support for improving glycaemic control in patients with type 2 diabetes: a meta-analysis of randomized controlled trials. BMC Public Health.

[32] Ruddock J, Poindexter M, Gary-Webb T, Walker E, Davis N (2016). Innovative strategies to improve diabetes outcomes in disadvantaged populations. Diabet Med.

[33] Shiyanbola OO, Ward E, Brown C (2018). “i did not want to take that medicine”: African-Americans’ reasons for diabetes medication nonadherence and perceived solutions for enhancing adherence. Patient Prefer Adherence.

[34] Shiyanbola OO, Ward EC, Brown CM (2018). Utilizing the common sense model to explore African Americans’ perception of type 2 diabetes: a qualitative study. PLoS One.

[35] Joseph DH, Griffin M, Hall RF, Sullivan ED (2001). Peer coaching: an intervention for individuals struggling with diabetes. Diabetes Educ.

[36] Philis-Tsimikas A, Fortmann A, Lleva-Ocana L, Walker C, Gallo LC. Peer-led diabetes education programs in high-risk Mexican Americans improve glycemic control compared with standard approaches: a Project Dulce promotora randomized trial. Diabetes Care. 2011:DC_102081. 10.2337/dc10-2081 PMC3161298 21775748

[37] Fisher WA, Fisher JD, Harman J. The information-motivation-behavioral skills model: a general social psychological approach to understanding and promoting health behavior. Soc Psychol Found Health Illness. 2003:82–106.

[38] Sabaté E (2003). Adherence to long-term therapies: evidence for action.

[39] Misovich SJ, Martinez T, Fisher JD, Bryan A, Catapano N (2003). Predicting breast self-examination: a test of the information-motivation-behavioral skills model 1. J Appl Soc Psychol.

[40] Fisher W, Fisher J, Harman J. The Information–Motivation–Behavioral skills model as a general model of health behavior change: theoretical approaches to individual-level change. Soc Psychol Found Health. 2003:127–53.

[41] Brown C, Battista DR, Bruehlman R, Sereika SS, Thase ME, Dunbar-Jacob J (2005). Beliefs about antidepressant medications in primary care patients: relationship to self-reported adherence. Med Care.

[42] Ross S, Walker A, MacLeod M (2004). Patient compliance in hypertension: role of illness perceptions and treatment beliefs. J Hum Hypertens.

[43] Horne R, Weinman J (2002). Self-regulation and self-management in asthma: exploring the role of illness perceptions and treatment beliefs in explaining non-adherence to preventer medication. Psychol Health.

[44] Hale ED, Treharne G, Kitas G (2007). The Common-Sense Model of self-regulation of health and illness: how can we use it to understand and respond to our patients’ needs?. Rheumatology..

[45] Shiyanbola OO, Maurer M, Ward E, Sharp LK, Lee J, Tarfa A. Protocol for partnering with peers intervention to improve medication adherence among African Americans with Type 2 Diabetes. medRxiv. 2020:2020.06.04.20122895.

[46] Mayberry LS, Gonzalez JS, Wallston KA, Kripalani S, Osborn CY (2013). The ARMS-D out performs the SDSCA, but both are reliable, valid, and predict glycemic control. Diabetes Res Clin Pract.

[47] Shiyanbola OO, Kaiser BL, Thomas GR, Tarfa A (2021). Preliminary engagement of a patient advisory board of African American community members with type 2 diabetes in a peer-led medication adherence intervention. Res Involve Engag.

[48] Shiyanbola OO, Tarfa A, Song A, Sharp LK, Ward E (2019). Preliminary feasibility of a peer-supported diabetes medication adherence intervention for African Americans. Health Behavior Policy Rev.

[49] Gearing RE, El-Bassel N, Ghesquiere A, Baldwin S, Gillies J, Ngeow E (2011). Major ingredients of fidelity: a review and scientific guide to improving quality of intervention research implementation. Clin Psychol Rev.

[50] Broadbent E, Petrie KJ, Main J, Weinman J (2006). The brief illness perception questionnaire. J Psychosom Res.

[51] Horne R, Weinman J, Hankins M (1999). The beliefs about medicines questionnaire: the development and evaluation of a new method for assessing the cognitive representation of medication. Psychol Health.

[52] Risser J, Jacobson TA, Kripalani S (2007). Development and psychometric evaluation of the Self-efficacy for Appropriate Medication Use Scale (SEAMS) in low-literacy patients with chronic disease. J Nurs Meas.

[53] Anderson RM, Funnell MM, Fitzgerald JT, Marrero DG (2000). The Diabetes Empowerment Scale: a measure of psychosocial self-efficacy. Diabetes Care.

[54] Weiss BD, Mays MZ, Martz W, Castro KM, DeWalt DA, Pignone MP (2005). Quick assessment of literacy in primary care: the newest vital sign. Ann Family Med.

[55] Fitzgerald JT, Anderson RM, Gruppen LD, Davis WK, Aman LC, Jacober SJ (1998). The reliability of the diabetes care profile for African Americans. Eval Health Prof.

[56] Lerman CE, Brody DS, Caputo GC, Smith DG, Lazaro CG, Wolfson HG (1990). Patients’ perceived involvement in care scale. J Gen Intern Med.

[57] Julious SA (2005). Sample size of 12 per group rule of thumb for a pilot study. Pharm Stat.

[58] Thabane L, Ma J, Chu R, Cheng J, Ismaila A, Rios LP (2010). A tutorial on pilot studies: the what, why and how. BMC Med Res Methodol.

[59] Gerber BS, Cano AI, Caceres ML, Smith DE, Wilken LA, Michaud JB (2010). A pharmacist and health promoter team to improve medication adherence among Latinos with diabetes. Ann Pharmacother.

[60] Turner DW (2010). Qualitative interview design: a practical guide for novice investigators. Qual Rep.

[61] Hsieh H-F, Shannon SE (2005). Three approaches to qualitative content analysis. Qual Health Res.

[62] Charmaz K, Belgrave L. Qualitative interviewing and grounded theory analysis. The SAGE handbook of interview research: the complexity of the craft. 2002;2(2002).

[63] Pope C, Ziebland S, Mays N. Qualitative research in health care: analysing qualitative data. BMJ. Br Med J. 2000;320(7227):114. 10.1136/bmj.320.7227.114 PMC1117368 10625273

[64] Richards L (2006). Handling qualitative data: an introduction.

[65] Birt L, Scott S, Cavers D, Campbell C, Walter F. Member checking a tool to enhance trustworthiness or merely a nod to validation? Qual Health Res. 2016. 10.1177/1049732316654870. 10.1177/1049732316654870 27340178

[66] Calvin D, Quinn L, Dancy B, Park C, Fleming SG, Smith E (2011). African Americans’ perception of risk for diabetes complications. Diabetes Educ.

[67] Rao D, Meyer J, Maurer M, Shiyanbola OO (2021). Perceptions of psychosocial and interpersonal factors affecting self-management behaviors among African Americans with diabetes. Explorator Res Clin Soc Pharm.

